# Prevalence of gestational diabetes mellitus and associated factors among women attending antenatal care at Gondar town public health facilities, Northwest Ethiopia

**DOI:** 10.1186/s12884-019-2492-3

**Published:** 2019-09-13

**Authors:** Achenef Asmamaw Muche, Oladapo O. Olayemi, Yigzaw Kebede Gete

**Affiliations:** 1 Pan African University Life and Earth Sciences Institute (including health and agriculture), Department of Obstetrics and Gynaecology, College of Medicine, University of Ibadan, Ibadan, Nigeria; 2 Department of Epidemiology and Biostatistics, Institute of Public Health, University of Gondar, Gondar, Ethiopia; 3 Department of Obstetrics and Gynaecology, College of Medicine, University College Hospital, University of Ibadan, Ibadan, Nigeria

**Keywords:** Gestational diabetes mellitus, Determinants, Overweight, Obesity, Physical activity, Dietary diversity, Antenatal depression

## Abstract

**Background:**

Globally, Gestational Diabetes Mellitus (GDM) is rising, but it is a neglected health threat to mothers and their children in low resource countries. Although, GDM is known in Ethiopia, information regarding it remains scarce by recent diagnostic criteria. Therefore, this study aimed to determine the prevalence of GDM and associated factors among women attending antenatal care at Gondar town public health facilities, Northwest Ethiopia.

**Methods:**

A cross-sectional study was conducted among 1027 pregnant women selected by the systematic random sampling technique. The universal one-step screening and diagnostic strategy was done using a two-hour 75 g oral glucose tolerance test. GDM was diagnosed using updated diagnostic criteria (2017 American Diabetes Association (ADA) or 2013 World Health Organization (WHO) or modified International Association of the Diabetes and Pregnancy Study Groups diagnostic criteria (IADPSG)). Binary logistic regression model was used to identify factors associated with GDM.

**Results:**

Of the total 1027 pregnant women, 12.8% (95% CI: 10.8–14.8) were diagnosed with GDM. Overweight and/or obesity (MUAC ≥28 cm) (AOR = 2.25, 95% CI: 1.18–4.26), previous history of GDM (AOR = 5.82, 95% CI: 2.57–13.18), family history of diabetes (AOR = 4.03, 95% CI: 1.57–10.35), low physical activity (AOR = 3.36, 95% CI: 1.60–7.04), inadequate dietary diversity (AOR = 1.9, 95% CI: 1.02–3.53), and antenatal depression (AOR = 4.12, 95% CI: 1.85–9.20) were significantly associated with GDM.

**Conclusions:**

The prevalence of GDM among women attending antenatal care at Gondar town public health facilities was high. Previous history of GDM, antenatal depression, family history of diabetes, low physical activity, overweight and/or obesity and inadequate dietary diversity were significantly associated with GDM. Routine screening of pregnant women and healthy lifestyle are strongly recommended.

## Background

The World Health Organization (WHO) defined “Gestational Diabetes Mellitus (GDM) as glucose intolerance first detected during pregnancy” [1]. Various adverse maternal and neonatal outcomes were complicated by GDM [2], while its complex care requires risk reduction strategies beyond the control of blood glucose level [3].

Globally, GDM affects an estimated 15% of the pregnant women, 87.6% of the hyperglycemia were in low and middle-income countries. It is one of the challenging health problems of sub-Saharan African countries [4].

A review indicated that the occurrence of GDM in sub-Saharan Africa was 14% [5] and Middle East and North Africa ranged from 8.4 to 24.5% [6] though the study used different screening and diagnostic criteria. Research findings also showed that the prevalence of GDM varied to a certain extent among regions in Africa. For example, East [7] and West [8] Africa reported 6 and 14%, respectively. Variations were also noted within sub-regions, like Rwanda [9] and Tanzania [10], where the prevalence was 8.3 and 19.5%, respectively. Two decades ago, the prevalence of GDM in the rural area of North Ethiopia was reported as 3.7% [11]. Additionally, a survey done in the same region (North Ethiopia) found a prevalence of 13% among urban women which was higher than that of women in rural areas (5%) [12]. However, the study used only fasting blood glucose test as diagnostic criteria for GDM.

According several studies, the increasing occurrence of GDM was related to advanced age, family history of diabetes, inactive physical activity, obesity, and risky behaviors [13–15]. Studies had also recognized that there was association between dietary habits during pregnancy and GDM. However, there had been no concrete consensus on the effects specific dietary aspects and the risk for GDM [16–18].

Gestational diabetes mellitus commonly identified during the second or third trimester of pregnancy as a result of the placental hormone plays an important role in the adverse effect on glucose metabolism [2]. As pregnancy progresses, various hormones such as estrogen, progesterone, leptin, cortisol, placental lactogen, and placental growth hormone promote a state of insulin resistance [19]. Primarily, human placental lactogen produced by placenta raises maternal blood glucose level and makes a woman’s body less sensitive to insulin leading to a higher-than-normal blood glucose level and perhaps GDM [20].

After the pre-existing diabetes (overt diabetes) is ruled out at the first antenatal visit, the recommended oral glucose tolerance test (OGTT) has to be performed at 24–28 weeks of pregnancy [21, 22]. However, since there has been no well-established national guideline for GDM screening in the country, most clinicians focus on risk factors to indicate GDM screening. This approach was leaving many pregnant women unnoticed until they develop symptoms of overt diabetes and complications.

Recognizing GDM offers an opportunity to reduce adverse pregnancy outcomes and improve the lifestyle to prevent the occurrence of diabetes in the future [23, 24]. Moreover, as per the knowledge of the principal investigator, no study has been conducted on prevalence of GDM in Ethiopia using the updated international diagnostic criteria. Therefore, the present study aimed to determine the prevalence of GDM and associated factors among women attending antenatal care at selected Gondar town public health facilities, Northwest Ethiopia.

## Materials and methods

### Study setting

The study was conducted at selected Gondar town public health facilities. It included one tertiary facility called University of Gondar Comprehensive Specialized Hospital (UOGCSH) and four health centers namely, Azezo, Gondar polyclinic, Woleka, and Maraki. Gondar town is located in the Northwestern part of Ethiopia, 747 km from Addis Ababa (the capital of Ethiopia), and 170 km from Bahirdar (the regional capital). It has 12 sub-cities with 12 urban and 10 rural kebeles (the smallest administrative units). Based on the 2014 population projection and census of the Central Statistical Agency (CSA) of Ethiopia [25], the total population of the town was estimated to be 306, 246 of whom 149, 970 were men and 156, 276 women. The majority (84.2%) of the people were Orthodox Christians, 11.8% Muslims and 1.1% Protestants. The expected number of pregnant women in the town was 11,225, of which at least 8913 were living in urban kebeles in 2017/18. The town had one comprehensive specialized hospital, eight health centers, and more than 15 private clinics.

### Study design, sample size, and sampling procedure

A cross-sectional study was conducted among a cohort of pregnant women receiving antenatal care at selected public health facilities from March 30, 2018 to January 4, 2019. The required sample size was determined using the single population proportion formula with the following statistical assumptions: a survey conducted by Management Sciences for Health (MSF) reported the prevalence of GDM among urban women in Tigray, Northern Ethiopia as 13% [12], a 95% confidence level, 3% marginal of error, a design effect of 2, and considering 15% of non-response and attrition rates.

### Applying the formula

$$ \mathrm{n}=\frac{{\left(\mathrm{z}\right)}^2\mathrm{P}\left(1\hbox{-} \mathrm{P}\right)}{{\mathrm{d}}^2}\kern1em n=\frac{(1.96)^2.13\left(1-.13\right)}{(0.03)^2}=482.7\kern1em \mathrm{n}=482.7\mathrm{x}2\kern0.5em \left(\mathrm{design}\kern0.5em \mathrm{effect}\right)=965 $$

Where, n = the sample size, Z = the desired level of the confidence interval, P = proportion of GDM, and d = margin of error. The minimum required sample size for significant result was 965, however we have added 15% by considered the non-response and attrition rates throughout the study period.

Therefore a total of 1110 were recruited for the study. A two-stage sampling technique used to invite pregnant mothers and include them in the cohort. On the first stage, one hospital and four health centers were selected by the simple random sample; on the second stage, pregnant women who fulfilled the inclusion criteria were chosen using the systematic random sampling technique.

### Participant selection and recruitment

During the study, 1110 new antenatal care attendants were invited and screened to participate in the study. Women were enrolled if they were aged 18 years or more with 20–23^+ 6^ weeks of gestational age and singleton pregnancy. But pregnant women who had pre-existing diabetes mellitus or overt DM, chronic diseases, medications that may affect glucose metabolism such as steroids, β-adrenergic agonists, anti-psychotic drugs [26, 27] were excluded. On their first visit, participants were asked for written consent for their enrolment in the study.

Screening at first visit was carried out according to the recommendations of the International Diabetes Federation (IDF) [22], ADA [28], and WHO [29] in order to rule out pre-existing diabetes. However, most pregnant women did not fast in their first visits. For subsequent tests, participants were informed to come fasting on their next appointments. Universal screening for GDM using a two-hour 75 g OGTT was performed for all pregnant women at 24–28 weeks of gestational age. Besides, 352(34.3%) women had at least one type of risk factors for GDM (pre pregnancy BMI ≥ 30 Kg/m^2^, MUAC ≥28 cm, age ≥ 35 years, previous macrosomia, glycosuria, history of GDM, family history of diabetes, previous poor pregnancy outcome or developed pregnancy-related complications) were advised to repeat the test at 32–36 weeks even if their OGTT results were negative at regular tests and GDM diagnosis ascertained by the second test.

## Data collection

### Demographic, obstetric, lifestyle, and anthropometric information

A structured and pretested questionnaire was developed and interviewer-administered to all study participants The questionnaire was prepared in English and then translated to Amharic (the local and national language) and then translated back to English by language experts to check its consistency (English version questionnaire attached as Additional file 1).

A face to face interview was employed to gather information on residence, age, last normal menstrual period (LNMP), marital status, religion, ethnicity, level of education, occupational status, average monthly income, family history of diabetes, previous history of GDM and birth weight of the previous child, behavioral and lifestyle characteristics, such as current exposure to alcohol, coffee, antenatal depressive symptoms, physical activity, and dietary diversity. Gestational age estimations were based on a reliable LNMP combined with first-trimester ultrasonography (if available). Additionally, socio-demographic information (parity, gravidity, gestational age (early fetal ultrasound result), obstetric history, medical history, hemoglobin) were retrieved from ANC cards.

The short form International Physical Activity Questionnaire (IPAQ) was used to assessed the physical activities that women do as part of their everyday lives [30]. The IPAQ was suitable for adults between 15 and 69 years of age and implemented in different countries. It was designed to assess specific types of activity such as walking, moderate and vigorous intensity activities done at work, as part of house and yard work, to get place to place, and in spare time for recreation, exercise or sport. Women were asked to recall their activities of the last 7 days preceding the interview. Data was reported as metabolic equivalents (MET-minutes per week) using the IPAQ scoring protocol to considered women into high, moderate and low level of physical activity categories [30].

Dietary diversity was assessed using a 24-h food recall method by the Food and Nutrition Technical Assistance (FANTA) 2016 version woman’s minimum dietary diversity measurement tool [31]. It contained a list of ten food groups (starchy staples, nuts and seeds, pulses, dairy, meat, eggs, poultry and fish, dark green leafy vegetables, other vitamin-A rich fruits and vegetables, other vegetables, and other fruits). The minimum dietary diversity score (MDDS) was dichotomized on the basis of whether or not women have consumed the list of defined food groups the previous day or night. The MDDS of five and more was categorized as adequate dietary diversity [31].

A mother was asked, “How often have you drunk coffee since your pregnancy?” If the answer was “daily” or “sometimes in a week” the mother was categorized as exposed to coffee. She was also asked, “How often have you drunk alcohol since your pregnancy?” If the answer was “daily” or “sometimes in a week” the mother was labelled as exposed to alcohol.

The mid-upper arm circumference (MUAC) was measured on the left arm using a non-stretchable measuring tape. As the result of most of the pregnant women could not recall their weight before conception, it was difficult to determine BMI. MUAC was as reliable measure due to quite stable during the course of pregnancy and highly correlated to the BMI before conception [32, 33]. Pregnant women with MUAC of ≥28 cm were considered as having overweight and/or obesity [34].

For measuring blood pressure (BP), the pregnant women were asked to take rest for at least 5 min in sitting positions if they were exerted. Then, the pressure was measured on the right arm using normal cuffs fitted for adults with a standard sphygmomanometer placing the stethoscope bell lightly over the brachial artery. The average systolic blood pressure (SBP) and diastolic blood pressure (DBP) were recorded in mmHg after two readings were taken with an intermission of 5–10 min. Hypertension was considered to be present if the systolic and diastolic blood pressures were greater than or equal to 140 mmHg and 90 mmHg, respectively.

Antenatal depression symptoms were measured by using the Edinburgh Postnatal Depression Scale (EPDS) screening tool developed [35] and validated in urban Ethiopia [36]. The tool was used to measure the feelings a mother had experienced in the past week. The tool contains ten specific questions with four Likert scale response options (most of the time, sometimes, not often, never), scored from 0 to 3 (a higher score indicated more depressive symptoms), which is simple to use, can be scored by simple addition. An EPDS score of 13 and more used like similar studies conducted in Ethiopia and abroad [37–39] to categorize the presence of antenatal depression.

### Laboratory assessment

Pregnant women scheduled for 2 h-75 g OGTT were called and reminded to complete an overnight fast for 8–12 h. Blood glucose level was analyzed using 5 μl capillary whole blood with HemoCue Glucose B-201^+^ (A¨ngelholm AB, Sweden). (The specifications of the HemoCue Glucose B-201^+^ glucometer attached as Additional file 2). Pregnant women were asked to sit and take rest before the first sample was taken using a finger prick at the side of the fingertip with a sterile lancet after cleaning in disinfectant and allowed to dry. A drop of blood was allowed to fill the cuvette after wiping away the first 2 or 3 drops of blood. Wipe off excess blood on the outside of the cuvette tip and place the filled cuvette in the cuvette holder. After 40–240 s the glucose value of the sample was displayed. The necessary precautions were taken during sample collection. Then, they were given a 75 g of glucose dissolved in 250 mL of water which they drank within 5 min under supervision. Capillary blood samples were taken again at 1 h and 2 h. The whole blood capillary values were converted to plasma venous values by multiplying a constant factor of 1.11 [40]. GDM diagnosis was made by using the 2017 American Diabetes Association (ADA) [28] or 2013 WHO [41] or modified International Association of the Diabetes and Pregnancy Study Groups (IADPSG) [42] diagnostic criteria. Similar values have been accepted by the WHO though slightly modified as a range [29]. The diagnosis of GDM is made when one or more of the values was met (fasting: ≥ 92 mg/dL, 1 h: ≥180 mg/dL; 2 h: ≥ 153 mg/dL). Hemoglobin analysis was also carried out by laboratory technologists in their respective health facilities. Pregnant women with hemoglobin concentration below 11 g/dl were considered as having anaemia [43].

### Statistical analysis

All data were entered into Epi Info™ 7 software, then exported to statistical package for the social sciences (SPSS) version 22.0 (IBM, NY, USA) for analysis. Descriptive statistics, like frequency, percentage, and mean, SD, and the range were used for the presentation of variables. Tables and figure were also used for data presentation. Categorical variables were expressed as proportions and chi-square analysis was performed to compare proportions. Student t-test was used to compare means for normally distributed variables. Binary logistic regression model was used to identify factors associated with GDM. Variables with *P-*value of ≤0.20 in the bivariate analysis were exported to the multivariate analysis to control the possible effect of confounders. Model goodness of fit test was checked by the Hosmer-and Lemeshow test (*P*-value = 0.953). Multicollinearity was checked by using the variance inflation factor (VIF). Adjusted Odds Ratio (AOR) with a 95% confidence level was estimated to show the strength of association, and a *P*-Value < 0.05 was used to declare statistical significance in the multivariate analysis.

## Results

### Socio demographic and clinical characteristics of pregnant mothers

Out of the 1110 pregnant women invited to participate in the study, 83 mothers (52 did not return for OGTT, 15 did not complete the tests, 9 were diagnosed with overt diabetes, 5 have a medical emergency, and 2 had an abortion before OGTT) were excluded. The characteristics of pregnant women who of included and excluded in the final analysis were similar in terms of key socio demographic variables such as residence (*P* = 0.835), maternal age (*P* = 0.0691), parity (*P* = 0.544), gravidity (*P* = 0.109), marital status (*P* = 0.734), MUAC (*P* = 0.08), employment status (*P* = 0.056). However women with complete data were on average attended secondary educational level and above and had higher income level than those who were excluded in the analysis.

The remaining 1027 women who participated and completed OGTT with overall response rate of 92.5% were included in the analysis. The mean age of the women was 27.22 (SD ± 5.24) years. Most of the participants (91%) lived in urban areas, 92% were married, and 60.5% unemployed. Five hundred and ninety-eight (58.2%) of women had secondary education and above. The mean of the MUAC was 24.77 (SD ± 3.08) cm, systolic blood pressure was 112.07 (SD ± 11.91) mmHg, and diastolic blood pressure was 71.91 (SD ± 10.22) mmHg. The mean hemoglobin level of the pregnant women with GDM and normal glucose level was 12.39 (SD ± 1.80) g/dl and 12.64 (SD ± 1.74) g/dl, respectively. Nearly half, 504 (49.1%) of the women were nulliparous. Sixty-two (6%) had a family history of DM (Table 1).

**Table 1:** Socio demographic and clinical characteristics of the study participants attending antenatal care at Gondar town public health facilities, Northwest Ethiopia: March 2018–January 2019 (*N* = 1027)

Variables	n (%) or mean ± SD			t/× ^2^	*P* value
	Total participants (*n* = 1027)	Normal (*n* = 896)	GDM (*n* = 131)		
Maternal age (years)	27.22 ± 5.241	26.69 ± 5.047	30.86 ± 5.109	8.827	< 0.001
< 25	323 (31.5)	304 (94.1)	19 (5.9)	61.108	< 0.001
25–29	366 (35.6)	335 (91.5)	31 (8.5)		
30–34	214 (20.8)	168 (78.5)	46 (21.5)		
≥ 35	124 (12.1)	89 (71.8)	35 (28.2)		
Marital status					
Married	945 (92)	831(87.9)	114 (12.1)	5.094	0.024
Single and others^a^	82 (8.0)	65 (79.3)	17 (20.7)		
Educational level					
Not formal education	200 (19.5)	168 (84)	32 (16.0))	5.472	0.065
Primary education	229 (22.3)	194 (84.7)	35 (15.3)		
Secondary education and above	598 (58.2)	534 (89.3)	64 (10.7)		
Employment status					
Unemployed	621 (60.5)	556 (89.5)	65 (10.5)	7.393	0.007
Employed	406 (39.5)	340 (83.7)	66 (16.3)		
Residence					
Urban	935 (91)	812 (86.8)	123 (13.2)	1.497	0.221
Rural	92 (9.0)	84 (91.3)	8 (8.7)		
Monthly income (birr)	3159.7 ± 2973.05	3155.17 ± 2952.66	3190.8 ± 3120.55	0.128	0.898
< 1500	236 (23)	203 (86)	33 (14)	1.641	0.650
1500–2499	257 (25)	230 (89.5)	27 (10.5)		
2500–3999	230 (22.4)	199 (86.5)	31 (13.5)		
≥ 4000	304 (29.6)	264(86.8)	40 (13.2)		
Family history of DM					
No	965 (94)	862 (89.3)	103 (10.7)	62.264	< 0.001
Yes	62 (6)	34 (54.8)	28 (45.2)		
Parity	0.92 ± 1.208	0.91 ± 1.227	1.02 ± 1.063	1.013	0.311
Nullipara	504 (49.1)	449 (89.1)	55 (10.9)	5.717	0.057
Primipara	277 (27)	243 (87.7)	34 (12.3)		
Multipara	246 (24)	204 (82.9)	42 (17.1)		
Gravidity	2.12 ± 1.354	2.09 ± 1.364	2.32 ± 1.267	1.838	0.066
Primigravida	439 (42.7)	395 (90)	44 (10)	5.146	0.023
Multigravida	588 (57.3)	501 (85.2)	87 (14.8)		
MUAC	24.77 ± 3.08	24.52 ± 2.837	26.47 ± 4.014	6.93	< 0.001
MUAC < 28 cm	852 (83)	767 (90)	85 (10)	34.700	< 0.001
MUAC ≥28 cm	175 (17)	129 (73.7)	46 (26.3)		
Blood pressure					
Systolic blood pressure (mmHg)	112.07 ± 11.91	111.57 ± 11.31	115.46 ± 15.01	3.508	< 0.001
Diastolic blood pressure (mmHg)	71.91 ± 10.22	71.60 ± 9.88	74.05 ± 12.09	2.565	0.010
Blood glucose level^b^					
Fasting blood glucose (mg/dL)	81.17 ± 12.960	78.3 ± 9.55	100.81 ± 15.88	22.779	< 0.001
1-h blood glucose (OGTT) (mg/dl)	140.69 ± 24.55	137.31 ± 22.0	163.82 ± 28.42	12.368	< 0.001
2-h blood glucose (OGTT) (mg/dl)	119.10 ± 19.75	116.22 ± 17.40	138.76 ± 23.40	13.188	< 0.001
Hemoglobin (g/dl)^c^	12.609 ± 1.75	12.64 ± 1.74	12.39 ± 1.80	−1.545	0.123
Normal (Hb ≥ 11 g/dl)	874 (86.6)	769 (88.0)	105 (12.0)	1.949	0.163
Anemia (Hb < 11 g/dl)	135 (13.4)	113 (83.7)	22 (16.3)		

*GDM* gestational diabetes mellitus, *h* hour, *t* student t value, *x*^2^ *chi square*, *Hb* hemoglobin, *g/dl* gram per deciliter, *mg/dl* milligram deciliter, *OGTT* oral glucose tolerance test, *MUAC* Mid upper arm circumference

^a^Divorced or widowed

^b^Blood glucose value at regular time (24–28 weeks)

^c^18 participants were missed (*N* = 1009)

### Behavioral and lifestyle characteristics of pregnant mothers

Out of total participants, a moderate level of physical activity was reported by 44.8%. Normal glucose level was more common among women having high physical activity level (33.4% vs 17.6%) and adequate dietary diversity (51.5% vs 27.5%) than women with GDM. Concerning exposure to drinking alcohol and coffee, no statistically significant difference was observed among women with normal glucose profile and GDM at *P* = 0.476 and *P* = 0.311 respectively. Eighty-nine (8.7%) of the pregnant women had antenatal depression symptoms (Table 2).

**Table 2:** Behavioral and life style characteristics of the study participants attending antenatal care at Gondar town public health facilities, Northwest Ethiopia: March 2018–January 2019 (*N* = 1027)

Variables	Total participants (*n* = 1027)	Normal (*n* = 896)	GDM (*n* = 131)	X^2^	*P* value
Level of physical activity					
High	322 (31.4)	299 (92.9)	23 (7.1)	100.841	< 0.001
Moderate	460 (44.8)	429 (93.3)	31 (6.7)		
Low	245 (23.9)	168 (68.6)	77 (31.4)		
Dietary diversity status					
Adequate (≥5)	497 (48.4)	461 (92.8)	36 (7.2)	26.294	< 0.001
Inadequate (< 5)	530 (51.6)	435 (82.1)	95 (17.9)		
Alcohol intake					
No	574 (55.9)	497 (86.6)	77 (13.4)	0.508	0.476
Yes	453 (44.1)	339 (88.1)	54 (11.9)		
Coffee intake					
No	291 (28.3)	249 (85.6)	42 (14.4)	1.027	0.311
Yes	736 (71.7)	647 (87.9)	89 (12.1)		
Antenatal depression					
No	938 (91.3)	839 (89.4)	99 (10.6)	47.128	< 0.001
Yes	89 (8.7)	57 (64)	32 (36)		

*GDM* Gestational Diabetes Mellitus, *x*^2^ *chi square*

### Obstetric history of pregnant mothers

Of the total of 588 pregnant women who had prior pregnancy history, 71(12.1%) had macrocosmic babies, 166 (28.2%) history of abortion, 38 (6.5%) history of stillbirth, 42 (7.1%) history of preterm labor, 97(16.5%) history of caesarean delivery, and 54 (9.2%) previous GDM (Table 3).

**Table 3:** Obstetric history of the study participants attending antenatal care at Gondar town public health facilities, Northwest Ethiopia: March 2018–January 2019 (*N* = 588)

Variables	Total participants (*n* = 588)	Normal (*n* = 502)	GDM (*n* = 86)	X^2^	*P* value
History of having macrocosmic baby					
No	517 (87.9)	453 (87.6)	64 (12.4)	17.309	< 0.001
Yes	71 (12.1)	49 (69)	22 (31)		
History of delivery by caesarean delivery					
No	491 (83.5)	423 (86.2)	68 (13.8)	1.437	0.231
Yes	97 (16.5)	79 (81.4)	18 (18.6)		
History of abortion					
No	422 (71.8)	368 (87.2)	54 (12.8)	4.007	0.045
Yes	166 (28.2)	134 (80.7)	32 (19.3)		
History of still birth					
No	550 (93.5)	476 (86.5)	74 (13.5)	9.351	0.002
Yes	38 (6.5)	26 (68.4)	12 (31.6)		
Preterm labor					
No	546 (92.9)	469 (85.9)	77 (14.1)	1.676	0.195
Yes	42 (7.1)	33 (78.6)	9 (21.4)		
Previous history GDM					
No	534 (90.8)	477 (89.3)	57 (10.7)	72.718	< 0.001
Yes	54 (9.2)	25 (46.3)	29 (53.7)		

*GDM* Gestational Diabetes Mellitus, *x*^2^ *chi square*

### Prevalence of GDM

**Fig. 1:**
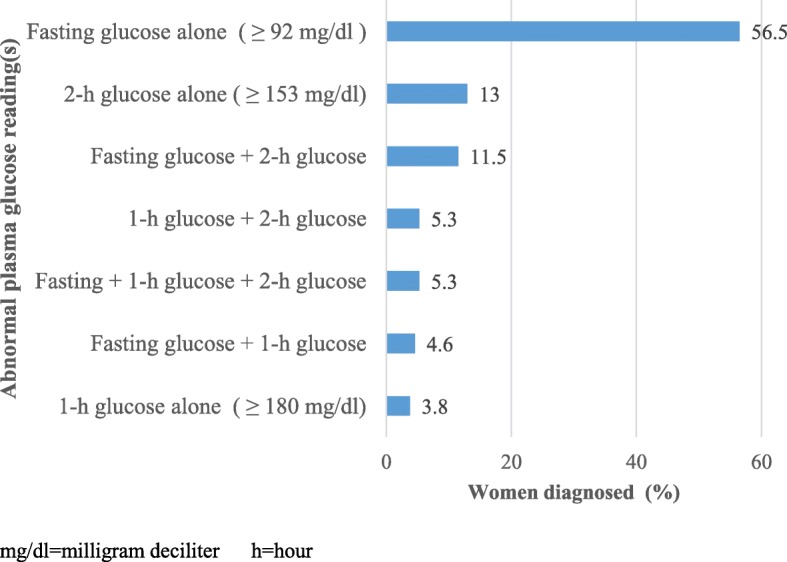
Analysis of the abnormal blood glucose reading among pregnant women with GDM attending antenatal care at Gondar town public health facilities, Northwest Ethiopia: March 2018–January 2019 (*N* = 131)

A total of 131 women were diagnosed as having GDM according to 2017 ADA and IADPSG diagnostic criteria which resulted in a GDM prevalence of 12.8% (95% CI: 10.7–14.8%); 118 (90%) of them were diagnosed at regular OGTT and the rest 13 (10%) at late OGTT. At regular OGTT test (24–28 weeks), the mean fasting, 1 h, and 2-h glucose levels of the pregnant women were 81.17 (SD ± 12.96) mg/dl, 140.69 (SD ±24.55) mg/dl, and 119.10 (SD ± 19.75) mg/dl, respectively. The plasma glucose levels taken at the three time points of the OGTT were significantly different for women with GDM and normal glucose level (*p* < 0.001). Pregnant women with GDM had the mean fasting, one-hour and two-hour glucose level of 100.81 (SD ±15.88) mg/dl, 163.82 (SD ± 28.42) mg/dl, and 138.76 (SD ±23.40) mg/dl, respectively. Whereas, women with normal glucose profiles had the mean fasting, one-hour and two-hour glucose levels of 78.3 (SD ± 9.55) mg/dl, 137.31 (SD ±22) mg/dl, and 116.22 (SD ±17.4) mg/dl, respectively (Table 1). More than half (56.5%) of the women with GDM were diagnosed on a fasting plasma glucose status only, while 21.4% had high fasting glucose level and one or two extra abnormal levels (Fig. 1).

### Factors associated with GDM

Results of the unadjusted binary logistic regression showed that advanced maternal age, marital status, level of education, employment status, MUAC ≥28 cm, parity, previous history of GDM, family history of DM, history of having macrocosmic baby, history of abortion, history of stillbirth, SBP, DBP, anemia (Hb < 11 g/dl), level of physical activity, dietary diversity, and antenatal depression were associated with GDM. However, on multivariate logistic regression, variables GDM was independently associated with being overweight and/or obese women (MUAC ≥28 cm) AOR = 2.25; 95% CI: 1.18–4.26), previous history of GDM (AOR = 5.82; 95% CI: 2.57–13.18), family history of diabetes (AOR = 4.03; 95% CI: 1.57–10.35), low physical activity (AOR = 3.36; 95% CI: 1.60–7.04), inadequate dietary diversity (AOR = 1.9; 95% CI: 1.02–3.53), and antenatal depression (AOR = 4.12; 95% CI: 1.85–9.20) were significantly associated with GDM (Table 4).

**Table 4:** Factors associated with GDM among pregnant women attending antenatal care at Gondar town public health facilities, Northwest Ethiopia: March 2018–January 2019

Variables	Non-GDM n (%)	GDM n (%)	COR (95% CI)	*P* -value	AOR (95% CI) ^a^	*P* value
Maternal age (years)						
< 25	304 (94.1)	19 (5.8)	1		1	
25–29	335 (91.4)	31 (8.5)	1.48 (0.82, 2.68)	0.194	0.99 (0.32, 3.12)	0.994
30–34	168 (78.5)	46 (21.5)	4.38 (2.49, 7.72)	< 0.001	2.24 (0.73, 6.83)	0.156
≥ 35	89 (71.8)	35 (28.2)	6.29 (3.43, 11.54)	< 0.001	3.05 (0.88, 10.51)	0.077
Marital status						
Married	831(87.9)	114(12.1)	1		1	
Single and others^b^	65(79.3)	17(20.7)	1.91 (1.08, 3.37)	0.026	2.04 (0.72, 5.78)	0.179
Educational level						
Not formal education	168 (84)	32 (16.0)	1		1	
Primary education	194 (84.7)	35 (15.3)	0.95 (0.56, 1.60)	0.838	1.82 (0.75, 4.43)	0.184
Secondary education and above	534 (89.3)	64 (10.7)	0.63 (0.40, .99)	0.048	0.90 (0.39, 2.11)	0.815
Employment status						
Unemployed	556 (89.5)	65 (10.5)	1		1	
Employed	340 (83.7)	66 (16.3)	1.66 (1.15, 2.40)	0.007	1.44 (0.71, 2.91)	0.309
MUAC						
MUAC < 28 cm	767 (90)	85 (10)	1		1	
MUAC ≥28 cm	129 (73.7)	46 (26.3)	3.22(2.15, 4.82)	< 0.001	2.25 (1.18, 4.26)	0.013
SBP (mmHg)	111.57 ± 11.31	115.46 ± 15.01	1.025(1.01, 1.04)	0.001	1.024 (0.99, 1.06)	0.132
DBP(mmHg)	71.60 + 9.882	74.05 ± 12.09	1.022 (1.01, 1.04)	0.011	0.99(0.96, 1.03)	0.757
Anemic status						
Normal (Hb ≥ 11 g/dl)	769 (88.0)	105 (12.0)	1		1	
Anemia (Hb < 11 g/dl)	113 (83.7)	22 (16.3)	1.43 (0.86, 2.35)	0.164	1.73 (0.81, 3.70)	0.160
Level of physical activity						
High	299 (92.9)	23 (7.1)	1		1	
Moderate	429 (93.3)	31 (6.7)	0.94 (.054, 1.64)	0.827	0.613 (0.27, 1.38)	0.238
Low	168 (68.6)	77 (31.4)	5.96 (3.60, 9.85)	< 0.001	3.36 (1.60, 7.04)	0.001
Dietary diversity status						
Adequate (≥5)	461 (92.8)	36 (7.2)	1		1	
Inadequate (< 5)	435 (82.1)	95 (17.9)	2.80 (1.86, 4.19)	< 0.001	1.90 (1.02, 3.53)	0.042
Family history of diabetes						
No	862 (89.3)	103 (10.7)	1		1	
Yes	34 (54.8)	28 (45.2)	6.89 (4.02, 11.83)	< 0.001	4.03 (1.57, 10.35)	0.004
Parity						
Nullipara	449 (89.1)	55 (10.9)	1		1	
Primipara	243 (87.7)	34 (12.3)	1.14 (0.72, 1.80)	0.567	0.77 (0.28, 2.16)	0.626
Multipara	204 (82.9)	42 (17.1)	1.68 (1.09, 2.60)	0.019	0.99 (0.34, 2.81)	0.987
History of macrocosmic baby						
No	453 (87.6)	64 (12.4)	1	1	1	
Yes	49 (69)	22 (31)	3.18 (1.80, 5.60)	< 0.001	1.54 (0.70, 3.37)	0.283
History of abortion						
No	368 (87.2)	54 (12.8)	1		1	
Yes	134 (80.7)	32 (19.3)	1.63 (1.01, 2.63)	0.047	1.014 (0.51, 2.03)	0.968
History of still birth						
No	476 (86.5)	74 (13.5)	1		1	
Yes	26 (68.4)	12 (31.6)	2.97 (1.44, 6.14)	0.003	1.42 (0.48, 4.15)	0.526
Previous GDM						
No	477 (89.3)	57 (10.7)	1		1	
Yes	25 (46.3)	29 (53.7)	9.71 (5.32, 17.71)	< 0.001	5.82 (2.57, 13.18)	< 0.001
Antenatal depression						
No	839 (89.4)	99 (10.6)	1		1	
Yes	57 (64)	32 (36)	4.76 (2.94, 7.69)	< 0.001	4.12 (1.85, 9.20)	0.001

*OR* odds ratio, *CI* confidence interval, *MUAC* mid-upper arm circumference, *SBP* systolic blood pressure, *DBP* diastolic blood pressure, 1 Reference

^a^Adjusted for factors including maternal age, marital status, educational level, employment status, MUAC, systolic blood pressure, diastolic blood pressure, anemia, level of physical activity, dietary diversity status, family history of diabetes, parity, history of macrocosmic baby, history of abortion, history of still birth, previous GDM and antenatal depression

^b^Divorced or widowed

## Discussion

This study was conducted to determine the prevalence of GDM and associated factors among women attending antenatal care at Gondar town public health facilities, Northwest Ethiopia, using the updated international diagnostic criteria.

In this study, the overall prevalence of GDM among women was 12.8% (95% CI: 10.8–14.8). The result was consistent with the Management Sciences for Health (MSF) reported the prevalence of GDM among urban women in Tigray, Northern Ethiopia (13%), but the study used only a fasting blood glucose test which means it did not consider internationally recommended diagnostic criteria [12]. The finding was higher than those of studies conducted in Rwanda (8.3%) [9], Tanzania (5.9%) [7], Egypt (8%) [44], and Nigeria (8.6%) [45]. The main reason for the high prevalence of GDM in this study setting might be the fact that the lower cut-off points for FPG and OGTT were used in the updated diagnostic criteria. On the other hand, the finding is lower than those of studies conducted in Tanzania (19.5%) [10] and South Africa (25.8%) [46] which used similar diagnostic criteria. This evidence indicates that the prevalence of GDM might also be affected not only by different diagnostic criteria but also by the characteristics of the population [2, 45, 46]. Increased testing for GDM, change in lifestyle, and the rising prevalence of overweight and obesity might have contributed [47].

Women with the mid-upper arm circumference of ≥28 cm were two times more likely to develop GDM than women with MUAC < 28 cm. This finding agreed with findings from Tanzania by Mwanri et al. [7] and Njete et al [10]. .Although, pre-gestational weight was not available in this study, different studies in Egypt, Ghana and South Africa which considered obesity using BMI noted its significant association with GDM [14, 15, 48]. Similarly, a review and meta-analysis by Nelson SM et al [49]. revealed that pre-pregnancy BMI was more strongly associated with the risk of GDM. This was happened by the fact that the decreased insulin sensitivity in obese pregnancies, resulted increases the blood glucose level [49, 50]. Overweight or obesity exposed to sedentary life led to obesity due to inactive lifestyle. This cycle also adversely affects the glucose metabolism. Likewise, we expect a further rise in GDM in the coming years as obesity is emerging as a public health problem.

Pregnant women with a previous history of GDM had six fold more increased odds of developing of the problem during the pregnancy at the moment. This finding was in line with those of studies in Egypt [48], Nigeria [51], Colorado [52], and a systematic review by Catherine Kim et al. [53]. The frequent occurrence of GDM showed the presence of communal risk factors in succeeding pregnancies [54]. Similarly, GDM was four fold higher in women with family history of diabetes. The finding was consistent with an evidence in the US [55] and studies in Iran [56, 57]. This might be the fact that the hyperglycemia was linked with a genetically dysfunction of beta cell and familial predisposition to insulin secretory defects [58]. Moreover, living standards and lifestyles of families are more likely similar resulting in sharing the related risk factors [59].

Low physical activity increases the likelihood of developing GDM at least three times compared to a high level of physical activity during pregnancy. This finding was in line with a study conducted in Vietnam [60], a meta-analysis by Tobias et al. [61], and review by Biase et al. [59]. Increased level of physical activity during pregnancy reduces glucose levels by preventing weight gain and enhancement of insulin sensitivity [13, 60, 62].

Pregnant women with inadequate dietary diversity had two times higher GDM than their counterparts. Healthy eating habits and the consumption of food from a variety of dietary groups during pregnancy contributes to the prevention of pregnancy-related complications [63]. Despite a piece of contradict evidence regarding the impact of dietary variability during pregnancy and developing GDM, studies revealed the association between dietary diversity and GDM [17, 64–66]. The possible reason for the association between inadequate dietary diversity and GDM might be the fact that the majority of women with GDM were classified as inadequate dietary diversity group. Similarly, a large proportion women relied on the monotonous food group in which cereals were the most common consumed food group. Their diets were likely to have been excessive in refined carbohydrates and sugars. On the other hand, dietary diversity varied across a range of factors related to the demographic and socio-economic status of individuals and households. Further research is required to know whether improving dietary pattern adherence during pregnancy is associated with a lower risk of GDM. Our result suggests that clinical and public health efforts to encourage dietary diversity for women of reproductive age might yield benefits in the reduction of GDM in future pregnancies.

Women with antenatal depression had four times higher GDM compared to women with non-depressive symptoms. The finding is supported studies by Morrison et al. [67] showed antenatal depression as a risk factor for GDM. The finding was in line with that of a study in Chicago which showed that a history of prenatal depression increased the risk for the development of GDM [68]. The association between depression and GDM was bidirectional [69] and could be explained by shared psychosocial and physiological factors for these comorbid situations. Pregnant women who had depression were more likely to practice unhealthy behaviors like sedentary lifestyle which lead to the risk of developing GDM [70]. Moreover, change in cortisol levels by depression have hyperglycemic effects [71, 72].

This study has strengths and limitation. The strength of the study was that it employed recent and universal screening tool to detect GDM and it has been done for all pregnant women at 24–28 weeks gestation. They underwent a two-hour 75 g OGTT, and updated standard reference cutoff values were considered. Besides, for pregnant women who had risk factors for GDM and whose OGTT results were negative at the regular test were tested again at late gestational age. WHO recommends that in settings where laboratories or proper storages and transport of blood samples is not guaranteed, which is the case in resource limited countries like Ethiopia, the use of point of care tests may influence the result [23]. However, we used plasma-calibrated hand-held glucometers because of convenience and acceptable reliability. Moreover, due to the nature of study design which restricted causal inference and could not reveal the temporal sequence between the factors and the outcome variable which could be limitation of the study.

## Conclusions

In conclusion, the overall prevalence of GDM was found to be high and a major public health concern among pregnant women in Gondar town, Northwest Ethiopia. Previous history of GDM, antenatal depression, family history of diabetes, low physical activity, overweight and/or obese women and inadequate dietary diversity were significantly associated with GDM. Given the imminent burden of obesity, unhealthy eating, and physical inactivity, a substantial threat of GDM is anticipated in Ethiopia. This may be an appropriate time to think about considering the need for routine screening of GDM to reduce the impact of the diseases in the country. In this regard, our study may serve as a baseline for larger studies and background evidence for policy debates for universal GDM screening in the existing health care system. Integrating healthy lifestyle choices into ANC services, such as reduced obesity, healthy eating, improvement in physical activities and effective strategies for coping with depression are strongly recommended.

## Supplementary information

**Additional file 1.** English version consent form and questionnaire for GDM survey among women attending ANC at Gondar town public health facilities, Northwest Ethiopia.

**Additional file 2.** The specifications of the HemoCue Glucose B-201+ glucometer.

## Data Availability

The datasets used and/or analyzed during the current study are available from the corresponding author on reasonable request.

## Abbreviations

ANC: Antenatal care
AOR: Adjusted odds ratio
BMI: Body mass index
BP: Blood pressure
CI: Confidence interval
CM: Centi meter
DBP: Diastolic blood pressure
DM: Diabetes mellitus
EDPS: Edinburgh Postnatal Depression Scale
FANTA: Food and Nutrition Technical Assistance
FPG: Fasting plasma glucose
GDM: Gestational diabetes mellitus
HAPO: Hyperglycemia and adverse pregnancy outcome
IADPSG: International Association of the Diabetes and Pregnancy Study Group
IDF: International diabetes federation
IGT: Impaired glucose tolerance
IPAQ: International Physical Activity Questionnaire
LMIC: Low and middle-income countries
LNMP: Last normal menstrual period
MDDS: Minimum dietary diversity score
MET: Metabolic equivalent of task
MUAC: Mid-upper arm circumference
NCD: Non-communicable diseases
OGTT: Oral glucose tolerance test
OR: Odds ratio
SBP: Systolic blood pressure
SD: Standard deviation
SPSS: Statistical package for social sciences
WHO: World Health Organization
