# Supplementary material for: Prevalence of gestational diabetes mellitus and associated factors among women attending antenatal care at Gondar town public health facilities, Northwest Ethiopia
Source: BMC Pregnancy Childbirth. 2019 Sep 13;19:334. doi: 10.1186/s12884-019-2492-3 (PMC6743162; doi:10.1186/s12884-019-2492-3)
Supplement: Supplementary file 1 — Additional file 1. English version consent form and questionnaire for GDM survey among women attending ANC at Gondar town public health facilities, Northwest Ethiopia. [file 12884_2019_2492_MOESM1_ESM.pdf]

Consent form for participation in research

Greetings.

Dear participant!

Hello! My name is ___________, I am here on behalf of **Mr. Achenef Asmamaw Muche,** a PhD student at Pan African University Life and Earth Sciences Institute, University of Ibadan, Ibadan, Nigeria. He is conducting a research for the partial fulfilment of Ph.D degree in Reprodcutive Health Science. This study obtained ethical approval from the institutional review board of University of Ibadan/University College Hospital and University of Gondar. He has also received permission from the Amhara public health institute and the health authorities of the study sites to conduct the study.

You are kindly requested to be included in the study, which will have importance in improving maternal and child health services. Identifying women with gestational diabetes mellitus provides an opportunity to improve pregnancy outcomes and women to make changes in their lifestyle to help prevent the development of diabetes later in life. I am asking you to participate in this study voluntarily. You are requested to give full history taking, clinical examinations, permitted some information retrieved from ANC cards, and also requested to come fasting on your appointment to give a blood sample for laboratory investigation (2h-75 g OGTT) with the standard care for screening and/or diagnosis of GDM used for this research project. If you are diagnosed with GDM immediately you will be linked (reffered) to health providers and get possible treatment options in the respective public health facilities. No information concerning you, as an individual will be passed to another individual or institution without your agreement. Your participation is voluntary and you have the right not to participate fully or partially. Moreover, you are allowed to ask questions and have received adequate clarification in a language you understand. If you agree to be included in the study, you are requested to confirm this with your signature below.

May I continue? Yes-----1 No----2

Having been well explained and informed of the intentions and benefits of the study, I voluntary to participate in the study.

Code of the participant: ____

Participant's signature: ___________________________date_____________________

Name of data collector: ______________________signature________date__________

Name of supervisor: ________________________ signature________date__________

A questionnaire prepared to assess the prevalence of gestational diabetes mellitus and associated factors among women attending antenatal care at Gondar town public health facilities, Northwest Ethiopia.

Date of interview: ---

Health facility name:---

Code of the mother: ---

Address of the mother: --

Name of data collector: ---------------------------------------

Date of appointment for next visit :---------------------------

Section 1: Socio-demographic characteristics

Now, I would like to ask you the Socio-demographic characteristics of about your self.

No.	Questions	Responses (alternative choices)	Code
101	How old were you at your last birthday?	1----------Years 2. Unknown	
102	What is your place of residence	1. Urban 2. Rural	
103	What is your religion?	1. Orthodox<br>2. Muslim<br>3. Protestant<br>4. Catholic<br>5. Other (specify)------------	
104	What is your ethnic group?	1. Amhara<br>2. Oromo<br>3. Tigrie<br>4. Other (specify -------------	
105	What is your current marital status?	1. Single<br>2. Married<br>3. Divorced<br>4. Widowed<br>5. Separated	
106	What level of schooling have you completed?	1. Not read and write<br>2. Read and write<br>3. Grade1-8<br>4. Grade 9-12<br>5. Certificate<br>6. Diploma<br>7. Degree and above	
107	What is your main occupation?	1. Housewife<br>2. Farmer<br>3. Government Employee<br>4. Private employee	

		5. Merchant<br>6. Daily laborer<br>7. Student<br>8. Other (Specify)------------	
108	How many children do you have?	----------- children	
109	What is the amount your monthly income?	----------- Eth birr	
**Section 2. Current pregnancy and obstetric history**<br>Now, I would like to ask you about the pregnancy and obstetric history that you have had currently.			
No.	Questions	Responses (alternative choices)	Code
201	Gestational age (wks) by	i.LMP-------GA----weeks+--days<br>ii.by ultrasound (if available)<br>GA----weeks+----days	
202	For how many times have you been live birth (parity)	------------------	
203	Including this pregnancy, for how many times have you been pregnant (gravida)	-------------------	
204	What was your weight during the first ANC visit (kg)	1. --------- Kg 2. Unknown	
205	Weight before conception (kg)	1. ----------- Kg 2. Unknown	
206	Have you had high blood pressure (hypretention)?	1. Yes 2. No	
**Section 3. Prior pregnancy and obstetric history**<br>Now, I would like to ask you about the pregnancy and obstetric history that you have had prevsiouly.			
No.	Questions	Responses (alternative choices)	Code
301	Birth weight of the new born (the most recent newborn)	---------Kg	
302	Have you ever given birth to large size (macrsomic) baby?	1. Yes 2. No	
303	Have you ever given birth to preterm?	1. Yes 2. No	
304	Have you ever had a baby by cesarean delivery?	1. Yes 2. No	
305	Have you had abortions and/ miscarriages?	1. Yes 2. No	
306	Have you had Still birth?	1. Yes 2. No	
307	Have you delivered a baby with congenital abnormality?	1. Yes 2. No	
**Section 4. General medical history (personal and family) and behaviour**<br>Now, I would like to ask you about the general medical history that you have had in your life.			
No.	Questions	Responses (alternative choices)	Code
**A. Perosnal history of diseases**			
401	Previous history of GDM	1. Yes 2. No	
402	History of high blood pressure	1. Yes 2. No	
**B. Family history of diseases**			

403	Diabetes melitus	1. Yes 2. No	
404	High blood pressure	1. Yes 2. No	
**C. Substance use**			
405	Have you ever taken any type of alcoholic drink? (Beer, wine, spirit, 'palm wine, otika etc)	1. Daily<br>2. Occasionally<br>3. Stopped after conception<br>4. Stopped before conception<br>5. No at all	
406	Have you ever taken cofeee drink?	1. Daily<br>2. Occasionally<br>3. Stopped after conception<br>4. Stopped before conception<br>5. No at all	

Section 5. Assessment of Minimum Dietary Diversity for Women (MDD-W)

The next question focuses the ten women dietary diversity or varieties of foods from the lists of food with one type or conjugates within 24 hours from yesterday 12:00 to today 12:00 o'clock**.**

**No.**	**Food group**	**Examples**	**Consumed Yes=1 No=0**
501	Grains/cereals , roots & tubers	Bread, pasta, rice, biscuit, cookies or dried food made from oats, maize, barley, wheat, millet, sorghum, and others. Any food made from teff (injera, pencake, porridge) cerifam, faffa (pre-processed baby foods) and other packed baby foods: Potatoe, boina/cassava, buila, kocho, beetroot, and other roots	
502	Pulses	Bean, pea, lentil	
503	nuts and seeds	Sesame, flax, sunflower, and nuts	
504	Dairy	Milk, cheese, yogurt or other milk products	
505	Meat, poultry and fish	Beef, lamb, goat, chicken, fish	
506	Eggs	Egg	
507	Dark green leafy vegetables	Kale/gomen, spinach, kosta, green pepper	
508	Other vitamin A rich fruits and vegetables	Pumpkin, carrot, papaya, mango, sweet potato	
509	Other vegetables	cucumber, tomato , Green pepper, Mushroom, Zucchini, Onion	
510	Other fruits	Orange, banana, Apple, Avocado, Guava, Lemon	
	**Total score**		

Section 6. Assessment of physical activities

International Phycial activity Questinare

We are interested in finding out about the kind of physical activities that women do as part of their every day lives. The questions will ask you about the time you spent being physically active in the last 7 days. Please answer each question even if you do not consider yourself be an active person. Please think about the activities you do at work, as part of your house and yard work, to get place to place, and in your spare time for recreation, exercise or sport. Now, I would like to ask you about the physical Activity in the past (1) week including today.

In the past seven days

<table>
<tr><th>No.</th><th>Questions</th><th>Responses (alternative choices)</th><th>Skip</th></tr>
<tr><td colspan="4">Think about all the vigorous activities that you did in the last 7 days. Vigorous physical activities refer to activities that take hard physical effort and make you breathe much harder than normal. Think only about those physical activities that you did for at least 10 minutes at a time.</td></tr>
<tr><td>601</td><td>During the last 7 days, on how many days did you do vigorous physical activities like heavy lifting, digging, aerobics, or fast bicycling?</td><td>1. _____ days per week
2. No vigorous physical activities</td><td>If No, Skip Q 603</td></tr>
<tr><td>602</td><td>How much time did you usually spend doing vigorous physical activities on one of those days?</td><td>1. _____ hours per day
2. _____ minutes per day
3. Don’t know/Not sure</td><td></td></tr>
<tr><td colspan="4">Think about all the moderate activities that you did in the last 7 days. Moderate activities refer to activities that take moderate physical effort and make you breathe somewhat harder than normal. Think only about those physical activities that you did for at least 10 minutes at a time.</td></tr>
<tr><td>603</td><td>During the last 7 days, on how many days did you do moderate physical activities like carrying light loads, bicycling at a regular pace, or doubles tennis? Do not include walking.</td><td>1. _____ days per week
2. No moderate physical activities</td><td>If No, Skip Q 605</td></tr>
<tr><td>604</td><td>How much time did you usually spend doing moderate physical activities on one of those days?</td><td>1. _____ hours per day
2. _____ minutes per day
3. Don’t know/Not sure</td><td></td></tr>
<tr><td colspan="4">Think about the time you spent walking in the last 7 days. This includes at work and at home, walking to travel from place to place, and any other walking that you might do solely for recreation, sport, exercise, or leisure.</td></tr>
<tr><td>605</td><td>During the last 7 days, on how many days did you walk for at least 10 minutes at a time?</td><td>1. _____ days per week
2. No walking</td><td>If No, Skip Q 607</td></tr>
<tr><td>606</td><td>How much time did you usually spend walking on one of those days?</td><td>1. _____ hours per day
2. _____ minutes per day
3. Don’t know/Not sure</td><td></td></tr>
<tr><td colspan="4">The last question is about the time you spent sitting on weekdays during the last 7 days. Include time spent at work, at home, while doing course work and during leisure time. This may include time spent sitting at a desk, visiting friends, reading, or sitting or lying down to watch television</td></tr>
<tr><td>607</td><td>During the last 7 days, how much time did you spend sitting on a week day?</td><td>1. _____ hours per day
2. _____ minutes per day
3. Don’t know/Not sure</td><td></td></tr>
</table>

Section 7. Assessment of Antenatal Depression			
**Feelings of depression (EPDS).** Tell us the way you have been feeling in the past (1) week including today. In the past seven days,			
No.	Questions	Response	Score
701	In the last week, have you been able to laugh and see the funny side of things?	As much as I always used to<br>Not as much as I used to<br>Certainly not as much as I used to<br>Not at all	0<br>1<br>2<br>3
702	In the last week, have you looked forward with enjoyment to things?	As much as I always used to<br>Rather less<br>Certainly less<br>Never looked forward	0<br>1<br>2<br>3
703	In the last week, have you blamed yourself unnecessarily when things went wrong?	Most of the time<br>Sometimes<br>Rarely<br>Never	3<br>2<br>1<br>0
704	In the last week, have you been anxious or worried for no good reason?	Most of the time<br>Sometimes<br>Not often<br>Never	0<br>1<br>2<br>3
705	In the last week, have you felt scared or panicky for no good reason?	Most of the time<br>Sometimes<br>Rarely<br>Never	3<br>2<br>1<br>0
706	In the last week, have things been getting on top of you?	Most of the time unable to cope<br>Sometimes unable<br>Mostly able<br>Coping as usual	3<br>2<br>1<br>0
707	In the last week, have you been so unhappy that you have had difficulty sleeping?	Most of the time<br>Sometimes<br>Rarely<br>Never	3<br>2<br>1<br>0
708	In the last week, have you felt sad or miserable?	Most of the time<br>Sometimes<br>Occasionally<br>Never	3<br>2<br>1<br>0
709	In the last week, have you felt so unhappy that you have been crying?	Most of the time<br>Sometimes<br>Occasionally<br>Never	3<br>2<br>1<br>0
710	In the last week, has the thought of harming yourself occurred to you?	Frequently<br>Sometimes<br>Not often<br>Never	3<br>2<br>1<br>0
	**Total score**		

Section 8. Anthropometric and clinical assessments		
801	Current weight weight (kg)	---------Kg
802	Height	---------Cm
803	Body mass index	-------- kg/m^2
804	MUAC	---------Cm
805	Glucose in the urine	
806	Hemoglobin level (mg/dl)	----------mg/dl
807	Blood pressure (systolic /diastolic)	Systolic blood pressure (mmHg)------------------<br>Diastolic blood pressure (mmHg)------------------

**808. Blood glucose test**			
Gestaitionl age	Intial visit (20-23^{+6} weeks)	Regualr OGTT (24-28 weeks)	Late OGTT (32-36 weeks)
Result	FPG ---------- mg/dl<br>RPG ---------- mg/dl	FPG --------------- mg/dl<br>1hr------------------mg/dl<br>2hr------------------mg/dl	FPG --------------- mg/dl<br>1hr------------------mg/dl<br>2hr------------------mg/dl

Comments:

Thank you for your participation!
