# Supplementary material for: Prevalence of gestational diabetes mellitus and associated factors among women attending antenatal care at Gondar town public health facilities, Northwest Ethiopia
Source: BMC Pregnancy Childbirth. 2019 Sep 13;19:334. doi: 10.1186/s12884-019-2492-3 (PMC6743162; doi:10.1186/s12884-019-2492-3)
Supplement: Supplementary file 2 — Additional file 2. The specifications of the HemoCue Glucose B-201+ glucometer. [file 12884_2019_2492_MOESM2_ESM.pdf]

HemoCue® Glucose 201+ System Specifications

Intended use: Quantitative determination of glucose in whole blood using a specially designed analyzer, the HemoCue® Glucose 201 DM Analyzer specially designed microcuvettes, the HemoCue Glucose 201 Microcuvettes. The quantitative determination of the instant blood glucose concentration in circulation supplements the clinical evidence in the diagnosis and treatment of the diabetic patient as well as in the monitoring of neonatal blood glucose levels. To establish HemoCue Glucose reference values and an intervention level, neonatal blood samples should be evaluated against a suitable laboratory method, taking into consideration the difference between whole blood and plasma reference values. The HemoCue Glucose 201 DM Analyzer with blood plasma conversion multiplies the measured whole blood glucose value by a factor of 1.11 and displays a plasma equivalent glucose result. The HemoCue Glucose 201 Microcuvettes are for In Vitro Diagnostic use only. The HemoCue Glucose 201 DM Analyzer is only to be used with HemoCue Glucose 201 Microcuvettes. For professional use only.

**Category**	**HemoCue® Glucose 201+ System specifications**
Principle	Modified glucose dehydrogenase in which the total amount of glucose is measured at the end point photometrically
Calibration	Factory calibrated and traceable to the ID GC-MS method, needs no further calibration and no coding
Sample Material	Capillary, venous or arterial whole blood including neonatal blood
Displayed Range	Whole blood values: 0-22.2 mmol/L (0-400 mg/dL)<br>Plasma equivalent values: 0-24.6 mmol/L (0-444 mg/dL)
Results	40-240 seconds
Sample Volume	Approximately 5 µL whole blood
Shelf Life	Vials and each individually wrapped microcuvette: 9 months from manufacturing
Package Size	Individually wrapped: 4 x 25 microcuvettes<br>Vial: 4 x 25 microcuvettes
Dimension	Analyzer: 160 x 85 x 43 mm (6.3 x 3.35 x 1.69 inches)
Weight	Analyzer: 350 g (0.77 pounds) including batteries
Storage Temperature	Analyzer: 0-50 °C (32-122 °F) Microcuvette: below 8 °C (46 °F), incl. freezer. Up to 3 days in room temperature.
Operating Temperature	18-30 °C (64-86 °F)
Operating Humidity	Up to 90 % relative humidity, non-condensing
Hematocrit Range	20%-70%
Altitude	Not affected by altitude
Power	AC adapter or 4 AA batteries
Quality Control	Built-in self test; system can be verified using liquid control
Data Management	HemoCue® Basic Connect software, store and view and transfer result and patient data
